# 

**Published:** 1899-04

**Authors:** 


### BOOKS RECEIVED.

The Ready-Reference Handbook of Diseases of the Skin. By George Thomas Jackson, M. D., Professor of Dermatology, Woman's Medical College of the New York Infirmary, and in the Medical Department of the University of Vermont; Chief of Clinic and Instructor in Dermatology, College of Physicians and Surgeons, New York. New (3d) edition. In one 12mo volume of 638 pages, with 75 illustrations and a colored plate. Cloth, $2.50 net. Philadelphia and New York: Lea Brothers & Co. 1899.

Proceedings of the American Medico-Psychological Association at the Fifty-fourth Annual Meeting, held at St. Louis, Mo., May 10–13, 1898. Published by the Association. Edited by the Secretary, Dr. C. B. Burr, Flint, Michigan.

A Compend of Human Physiology. Especially Adapted for the Use of Medical Students. Quiz Compends, No. 4. By Albert P. Brubaker, A. M., M.D., Adjunct Professor of Physiology and Hygiene in the Jefferson Medical College; Professor of Physiology in the Pennsylvania College of Dental Surgery, etc., etc. Ninth edition, with new illustrations. Price 80 cents. Philadelphia: P. Blakiston's Son & Co. 1899.

Nervous and Mental Diseases. By Archibald Church, M. D., Professor of Clinical Neurology, Mental Diseases and Medical Jurisprudence, Northwestern University Medical School, Chicago: and Frederick Peterson, M. D., Clinical Professor of Medical Diseases, Woman's Medical College, New York. With 305 illustrations. Royal octavo, pp. 843. Cloth, $5.00; half morocco, $6.00. Philadelphia: W. B. Saunders. 1899.

Diseases of the Ear, Nose and Throat, and Their Accessory Cavities. By Seth Scott Bishop, M. D., D. C. L., LL. D., Professor of Diseases of the Nose, Throat and Ear in the Illinois Medical College; Professor in the Chicago Post-Graduate Medical School and Hospital; Surgeon to the Post-Graduate Hospital; one of the Editors of the *Laryngoscope*, etc. Second edition. Thoroughly revised and enlarged. Illustrated with 94 chromo-lithographs and 215 half-tone and photo-engravings; pp. xix.—554. Extra cloth, $4.00 net; sheep or half-Russia, $5.00 net. Philadelphia: The F. A. Davis Co. 1899.

A Text-Book on Practical Obstetrics. By Egbert H. Grandin, M. D., Gynecologist to the Columbus Hospital; Consulting Gynecologist to the French Hospital; Late Consulting Obstetrician and Obstetric Surgeon of the New York Maternity Hospital; Fellow of the American Gynecological Society, etc. With the collaboration of George W. Jarman, M. D., Gynecologist to the Cancer Hospital; Instructor in Gynecology in the Medical Department of the Columbia University; Late Obstetric Surgeon of the New York Maternity Hospital; Fellow of the American Gynecological Society, etc. Second edition; revised and enlarged. Illustrated with 64 full-page photographic plates and 86 illustrations in the text; pp. xiv.—461. Extra cloth, $4.00 net; sheep, $4.75 net. Philadelphia: The F. A. Davis Co. 1899.

A Handbook of Obstetric Nursing; Comprising the Course of Instruction in Obstetric Nursing given to the Pupils of the Training School for Nurses connected with the Woman's Hospital of Philadelphia. By Anna M. Fullerton, M. D., Demonstrator of Obstetrics in the Woman's Medical College of Pennsylvania; Physician-in-Charge and Obstetrician and Gynecologist to the Woman's Hospital of Philadelphia, and Superintendent of the Nurses Training School of the Woman's Hospital of Philadelphia. Forty illustrations; 12mo. Handsome cloth, $1.00. Fourth edition, revised. Philadelphia: P. Blakiston's Son & Co. 1899.

Diagnosis by the Urine, or the Practical Examination of Urine with Special Reference to Diagnosis. By Allard Menninger, M. D., Professor of Chemistry, Urinology and Hygiene in the Medical College of the State of South Carolina; Visiting Physician to the City Hospital of Charleston, etc., etc. Second edition, enlarged and revised. With illustrations. Philadelphia: P. Blakiston's Son & Co. 1899.

Addresses and Papers by the Members of the Instructing Staff of the New York State Veterinary College, at Cornell University, for the years 1896–98. Ithaca, N. Y.: Published by the University. 1898.

International Clinics. A Quarterly of Clinical Lectures and Specially Prepared Articles on Treatment and Drugs. By professors and lecturers in the leading medical colleges of the United States, Germany, Austria, France, Great Britain and Canada. Edited by Judson Daland, M. D. (Univ. of Penna.), Philadelphia, Instructor in Clinical Medicine and Lecturer on Physical Diagnosis in the University of Pennsylvania; Assistant Physician to the Hospital of the University of Pennsylvania, etc., etc. J. Mitchell Bruce, M. D., F. R. C. P., London, England, Physician to and Lecturer on the Principles and Practice of Medicine at the Charing Cross Hospital. David W. Finley, M. D., F. R. C. P., Aberdeen,

Scotland, Professor of Practice of Medicine in the University of Aberdeen; Physician to and Lecturer on Clinical Medicine in the Aberdeen Royal Infirmary, etc. Volume IV. Eighth Series. 1899. Octavo, pp. xii.—375. Philadelphia: J. B. Lippincott Co. 1899.

Progressive Medicine. A Quarterly Digest of Advances, Discoveries and Improvements in the Medical and Surgical Sciences. Edited by Hobart Amory Hare, M. D., Professor of Therapeutics and Materia Medica in the Jefferson Medical College of Philadelphia. Octavo, handsomely bound in cloth, 490 pages, 28 illustrations and 3 colored plates. Philadelphia and New York: Lea Brothers & Co.

